# Mitochondrial dysfunction in sepsis-associated acute kidney injury: mechanisms and therapeutic potential

**DOI:** 10.3389/fimmu.2026.1829288

**Published:** 2026-05-19

**Authors:** Chong Wang, Qi Liu, He Wang, Qian Zhang, Boxin Yang, Hongchao Liu, Zhongxin Li, Zhongjun Shen, Jingjin Tao, Zhen Xu, Yuying Nie, Xiangyi Xu, Huike Guo, Shuo Yang, Liyan Cui

**Affiliations:** 1 Department of Laboratory Medicine, Peking University Third Hospital, Beijing, China; 2 Core Unit of National Clinical Research Center for Laboratory Medicine, Peking University Third Hospital, Beijing, China

**Keywords:** apoptosis, ferroptosis, immunometabolism, metabolic reprogramming, mitochondrial dysfunction, oxidative stress, sepsis-associated acute kidney injury, therapeutics

## Abstract

Sepsis-associated acute kidney injury (SA-AKI) is a life-threatening complication in critically ill adult patients, accounting for nearly 50% of acute kidney injury (AKI) cases in intensive care units and carrying a mortality rate exceeding 40%. Its pathogenesis extends beyond traditional concepts of renal hypoperfusion to encompass a complex interplay of systemic inflammation, microcirculatory dysfunction, and profound metabolic reprogramming. Converging evidence now positions mitochondrial dysfunction as a central hub that integrates these pathogenic insults, ultimately driving tubular epithelial cell injury and renal functional decline. Importantly, mitochondrial dysfunction interfaces with innate immune activation (e.g., the mtDNA-cGAS-STING pathway) and immunometabolic reprogramming in both renal parenchymal and immune cells. This highlights mitochondria-immune crosstalk as a key determinant of SA-AKI pathogenesis. This review systematically examines the multidimensional nature of mitochondrial impairment in SA-AKI, including bioenergetic failure, disrupted fusion-fission dynamics, compromised quality control mechanisms, and aberrant redox signaling. We further explore the therapeutic potential of targeting mitochondrial pathways, critically assessing emerging strategies and their translational challenges, and discuss future directions for developing mechanism-based diagnostics and targeted therapies for this devastating syndrome.

## 1 Introduction

Acute kidney injury (AKI) affects over 13 million people annually worldwide, imposing a substantial burden on healthcare systems (1). Among its various etiologies, sepsis stands out as a leading and particularly devastating cause (2). Sepsis-associated acute kidney injury (SA-AKI), defined as AKI meeting Kidney Disease: Improving Global Outcomes(KDIGO) criteria (**Table 1**) in the setting of sepsis without other significant contributing factors, occurs in nearly half of septic patients and is associated with a dramatic increase in mortality (3, 4).

**Table 1:** KDIGO clinical practice guideline for acute kidney injury.

Stage	Serum creatinine criteria	Urine output criteria
1	Increase to 1.5–1.9 times baseline OR Increase by ≥0.3 mg/dl (≥26.5 µmol/L) within 48 hours	<0.5 mL/(kg·h) for 6–12 hours
2	Increase to 2.0–2.9 times baseline	<0.5 mL/(kg·h) for ≥12 hours
3	Increase to 3.0 times baseline OR Scr ≥4.0 mg/dl (≥353.6 µmol/L) OR Initiation of renal replacement therapy (RRT)	<0.3 mL/(kg·h) for ≥24 hours OR Anuria for ≥12 hours

Clinical differentiation of SA-AKI from other forms of AKI is essential for both diagnosis and management. Although KDIGO criteria (**Table 1**) define AKI based on serum creatinine and urine output, they do not distinguish between etiologies. In clinical practice, SA-AKI is diagnosed when AKI occurs in a patient with documented or suspected sepsis, after excluding other major causes of AKI, including: ischemia-reperfusion injury (e.g., following cardiac surgery, shock of non-septic origin, or renal artery occlusion); nephrotoxic agents (e.g., contrast media, aminoglycosides, vancomycin, non-steroidal anti-inflammatory drugs, or cisplatin); glomerulonephritis or interstitial nephritis (e.g., due to autoimmune diseases, drugs, or infections other than sepsis); and obstructive uropathy (e.g., calculi, tumors, or catheter obstruction). The clinical context is paramount: the presence of systemic inflammatory response syndrome (SIRS), documented infection, or positive blood cultures supports sepsis as the trigger, while the absence of alternative explanations (e.g., no recent nephrotoxin exposure, no hemodynamic collapse of non-septic cause, no evidence of post-renal obstruction) strengthens the diagnosis of SA-AKI. Biomarkers such as procalcitonin (PCT), presepsin, and urinary biomarkers (e.g., TIMP-2, IGFBP-7) may aid in differentiating septic from non-septic AKI, but none are fully specific. Therefore, SA-AKI remains a diagnosis of exclusion based on a combination of clinical judgment, laboratory data, and imaging when indicated. This pragmatic approach is reflected in the KDIGO consensus definition and in major clinical guidelines (4).

**Figure 1:**
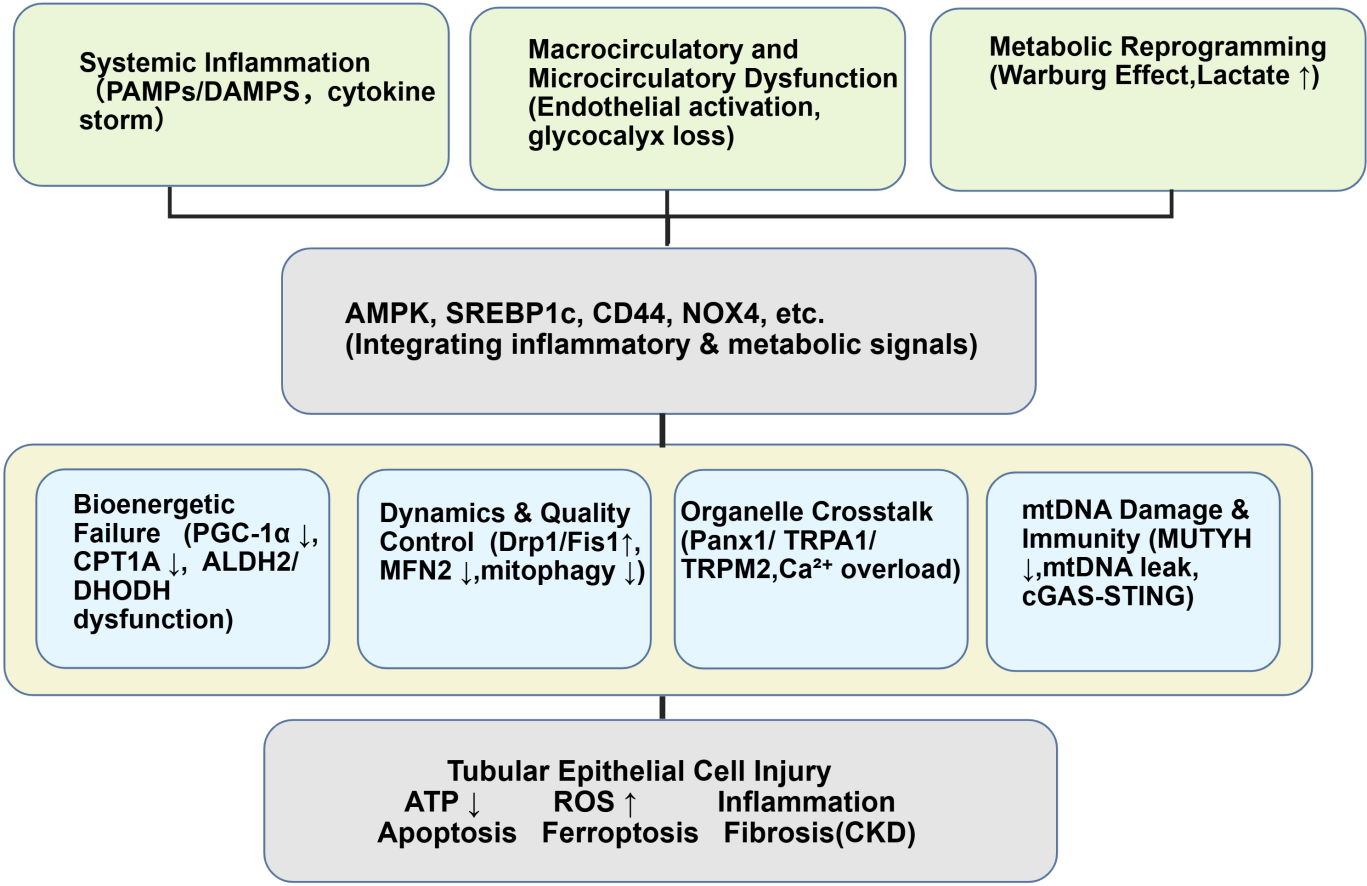
Multidimensional mitochondrial dysfunction in SA-AKI. Schematic overview illustrating the central role of mitochondrial dysfunction in SA-AKI. Systemic factors (inflammation, hemodynamic changes, metabolic reprogramming, RAAS dysregulation) converge on upstream signaling hubs, leading to dysfunction across multiple mitochondrial dimensions: bioenergetic failure, dynamics imbalance, quality control collapse, organelle crosstalk disruption, and mtDNA-mediated innate immune activation. These collectively drive tubular epithelial cell injury and progression to CKD. The **Figure 1** was created with BioGDP (9).

The pathogenesis of SA-AKI is complex and multifactorial (5). While traditionally attributed to renal hypoperfusion secondary to hemodynamic instability, it is now clear that SA-AKI can occur independently of systemic hemodynamic compromise (6). Contemporary understanding implicates an intricate interplay of systemic inflammation, microcirculatory dysfunction, and profound metabolic reprogramming (7). Within this framework, cellular and subcellular events—particularly mitochondrial dysfunction—have emerged as critical drivers of tubular injury and functional decline (8). As illustrated in **Figure 1**, mitochondrial impairment serves as a central hub integrating diverse pathogenic insults.

In this review, we synthesize current knowledge on the multifaceted nature of mitochondrial pathophysiology in SA-AKI. We systematically examine key dimensions of mitochondrial dysregulation: upstream triggers and signals, dynamics imbalance, quality control disorders, mtDNA damage and innate immunity, and disruptions in ion homeostasis and organelle interactions. Importantly, we position mitochondrial dysfunction within the broader context of mitochondria-immune crosstalk. Damaged mitochondria release mtDNA, ATP, and formyl peptides, which act as damage-associated molecular patterns (DAMPs) to activate pattern recognition receptors on both tubular cells and infiltrating immune cells. The mtDNA-cGAS-STING axis exemplifies this bidirectional communication: mitochondrial damage triggers innate immune signaling, which in turn amplifies mitochondrial dysfunction through cytokine-mediated ROS production and metabolic reprogramming. Furthermore, immune cell metabolism, especially the shift toward aerobic glycolysis in activated macrophages and T cells, competes with renal parenchymal cells for metabolic substrates. This generates a pro-inflammatory microenvironment that exacerbates tubular mitochondrial injury. Recognizing SA-AKI as a disease of disrupted mitochondria-immune homeostasis, we integrate this perspective throughout the review. Building on this mechanistic foundation, we critically evaluate emerging therapeutic strategies aimed at preserving or restoring mitochondrial homeostasis, ranging from direct mitochondrial-targeted agents to interventions that modulate upstream regulatory pathways. We also discuss translational challenges and future directions, highlighting the potential integration of mitochondrial health assessment into the diagnostic and therapeutic framework for SA-AKI.

## 2 Pathophysiological overview of SA-AKI

The pathogenesis of SA-AKI involves a complex, multifactorial interplay between systemic disturbances and intrinsic renal responses (8). While historically attributed primarily to renal hypoperfusion secondary to septic shock, contemporary understanding recognizes that SA-AKI arises from multiple pathways (6). Current research centers on several key mechanisms, including systemic inflammation, macrocirculatory and microcirculatory dysfunction, metabolic reprogramming, renin-angiotensin-aldosterone system (RAAS) dysregulation, and mitochondrial dysfunction, among others (7). These interconnected factors, which will be detailed in the following sections, collectively contribute to the initiation and progression of renal injury (10).Critically, the inflammatory cytokines and reactive oxygen species (ROS) generated during this process directly impair mitochondrial oxidative phosphorylation, disrupt electron transport chain complexes, and promote mitochondrial DNA (mtDNA) damage, establishing a feed-forward loop wherein mitochondrial dysfunction further amplifies the inflammatory response (see Section 2.5).

### 2.1 Systemic inflammation and immune dysregulation

Systemic inflammation serves as the initiating trigger in SA-AKI (8). Rather than a simple “cytokine storm,” the injury is inflicted through a complex cascade of events that directly and indirectly compromise renal function. Key intermediates in this process include immune cell infiltration, endothelial activation, coagulation abnormalities, and microcirculatory disturbances (11).

The inciting septic insult induces massive release of pathogen-associated molecular patterns (PAMPs) and damage-associated molecular patterns (DAMPs), which activate pattern recognition receptors (PRRs) on both immune and parenchymal cells (12). This activation orchestrates a cytokine storm characterized by elevated levels of tumor necrosis factor-α (TNF-α), interleukin-1β (IL-1β), and interleukin-6 (IL-6) (5). The resultant systemic inflammatory milieu exerts direct cytotoxic effects on renal cells and also propagates endothelial activation, leukocyte recruitment, and microvascular dysfunction. These events collectively create a hostile microenvironment that predisposes to tubular injury and mitochondrial dysfunction (10).

### 2.2 Macrocirculatory and microcirculatory dysfunction

Macrocirculatory and microcirculatory dysfunction are now recognized as central events in SA-AKI pathogenesis (13). Sepsis-induced systemic vasodilation and decreased vascular resistance traditionally were thought to cause renal hypoperfusion. However, accumulating evidence shows that kidney injury frequently develops independently of systemic hemodynamic compromise (13). Current understanding emphasizes microcirculatory dysfunction—characterized by endothelial activation, glycocalyx shedding, impaired capillary perfusion, and heterogeneous blood flow—as the critical link connecting systemic inflammation to tubular damage (14).This microcirculatory failure creates a microenvironment of local hypoxia, nutrient deprivation, and oxidative stress, directly compromising the function of highly metabolically active renal tubular cells and their mitochondria (15, 16). Direct evidence linking glycocalyx shedding products (e.g., syndecan-1) to tubular mitochondrial dysfunction is currently lacking, and this represents an important direction for future investigation.

### 2.3 Metabolic reprogramming

Metabolic reprogramming has emerged as a hallmark of SA-AKI, reflecting a fundamental shift in cellular energy metabolism (17). In response to septic stress, both infiltrating inflammatory cells and renal parenchymal cells undergo a metabolic transition from oxidative phosphorylation to aerobic glycolysis—a phenomenon akin to the Warburg effect (18). The functional significance of this shift in highly oxidative proximal tubular cells, however, remains debated. Current evidence suggests a dual nature. On one hand, the shift to glycolysis is proposed as a defense or tolerance mechanism that allows tubular epithelial cells to survive septic stress in the absence of overt necrosis or apoptosis, prioritizing cell survival over full functional output (8). In this view, metabolic reprogramming away from fatty acid oxidation and oxidative phosphorylation is an adaptive response to mitochondrial damage (17). On the other hand, this adaptation comes at a cost. Prolonged reliance on glycolysis fails to meet the high energy demands of proximal tubules and may contribute to tubular atrophy, fibrosis, and progression to chronic kidney disease (7, 10). Moreover, the metabolic shift generates a pro-inflammatory microenvironment that can exacerbate mitochondrial dysfunction (7, 19). Thus, the metabolic shift toward glycolysis in SA-AKI appears to be a double-edged sword: protective in the short term but potentially harmful if sustained. This sets the stage for the profound mitochondrial impairments discussed in Section 2 (7).

### 2.4 RAAS dysregulation

The RAAS plays a dual role in SA-AKI. Initially, its activation represents a compensatory response aimed at maintaining hemodynamic stability and glomerular filtration pressure during septic shock. However, this acute adaptation frequently transitions into sustained, maladaptive overactivation (20).

Persistent RAAS activation exacerbates early hemodynamic instability and intrarenal hypoxia through potent vasoconstriction of the efferent arteriole. More importantly, it drives pro-inflammatory and pro-fibrotic signaling cascades—primarily via angiotensin II (Ang II) and its type 1 receptor (AT1R)—that extend beyond hemodynamic effects (20). These pathways promote oxidative stress, endothelial dysfunction, and tubular injury, creating a microenvironment that indirectly but profoundly compromises mitochondrial homeostasis. Mechanistically, Regarding the access of Ang II to AT1R on tubular cells during sepsis, three complementary mechanisms are relevant. First, AT1R is expressed not only on the basolateral membrane but also on the apical (luminal) membrane of proximal tubular cells, where it can be accessed by Ang II present in the tubular fluid independent of peritubular capillary flow (21). Second, intrarenal Ang II generation is markedly enhanced in sepsis, leading to high local concentrations that can activate both basolateral and apical receptors (20). Third, ligand-bound AT1R undergoes endocytosis and continues to signal from intracellular compartments, sustaining downstream effects even under compromised peritubular perfusion (22). These mechanisms collectively ensure AT1R-mediated signaling in tubular epithelial cells during SA-AKI.

This maladaptive RAAS activation is now recognized as a core molecular driver of the transition from SA-AKI to chronic kidney disease (CKD), highlighting it as a potential therapeutic target not only for acute injury but also for long-term renal outcomes (23).

## 3 Multidimensional mitochondrial dysfunction in SA-AKI

Mitochondrial impairment in SA-AKI is not a singular defect but rather a syndrome encompassing multiple interdependent dysfunctions. The proximal tubule, with its dense mitochondrial network dedicated to active transport, is exquisitely vulnerable to these disturbances.

Integrated proteomic and metabolomic analyses have confirmed that mitochondrial dysfunction is a core pathological feature of SA-AKI, revealing significant downregulation of proteins involved in the respiratory chain, oxidative phosphorylation, and ATP metabolism in the kidneys of septic mice. Gene set enrichment analysis (GSEA) further corroborates these findings, positioning mitochondrial impairment at the center of SA-AKI pathogenesis (24).

Importantly, this dysfunction extends far beyond insufficient ATP production. It encompasses a complex web of pathogenic mechanisms, including ischemia- or toxin-induced mitochondrial damage, excessive reactive oxygen species (ROS) generation, and bioenergetic failure (25). Moreover, mitochondrial dysfunction and oxidative stress are now recognized as key drivers not only of AKI but also of its progression to CKD (10).

In the following sections, we systematically dissect the multidimensional nature of mitochondrial injury in SA-AKI, examining the interconnected axes of metabolic failure, dynamics imbalance, quality control disruption, organelle crosstalk, and mtDNA-mediated innate immunity.

### 3.1 Upstream triggers and signals

Mitochondrial dysfunction in SA-AKI is not an isolated event but rather the consequence of upstream signaling networks that sense and integrate inflammatory, metabolic, and oxidative stressors. Several molecular hubs have been identified that transduce these pathogenic insults into mitochondrial damage.

#### 3.1.1 AMP-activated protein kinase

AMPK serves as a master metabolic sensor linking inflammation to mitochondrial homeostasis. In SA-AKI, AMPK activation exerts protective effects by suppressing NF-κB-mediated inflammation and reversing pathogenic metabolic reprogramming through enhanced fatty acid oxidation (FAO). Mechanistically, AMPK activates the Sentrin-specific protease 1-Sirtuin 3 (SENP1-Sirt3) axis, leading to reduced mitochondrial ROS, restored Adenosine Triphosphate(ATP) levels, and attenuated tubular injury. Metformin, an AMPK agonist, has shown promise by engaging this pathway (26).However, its clinical application in SA-AKI must consider the risk of lactic acidosis, particularly in patients with hypoperfusion or renal impairment. Detailed safety considerations, contraindications, and monitoring requirements are provided in **Table 2**.

**Table 2:** Emerging therapeutic strategies targeting mitochondrial dysfunction in SA-AKI.

Target/pathway	Intervention strategy	Mechanism of action	Experimental model	Main effect	Stage and key points	Clinical translatability (status, barriers, applicability)
AMPK (26)	Metformin	Activate SENP1−Sirt3 axis; Inhibition of NF-κB; Restore FAO	CLP mice; LPS cells	ROS ↓, ATP ↑, apoptosis ↓, mitochondrial function ↑	Clinically marketed (requiring new indication research). Advantages: approved drug, clear safety. Challenges: renal targeting needed	Status: Phase IV (diabetes), no SA−AKI trial (preclinical only), low evidence. Barriers: renal tubular delivery, lactic acidosis risk in shock. Applicability: early SA−AKI with preserved perfusion; not in hypoperfusion/shock. Contraindicated in septic shock and eGFR <45 mL/min/1.73 m²; close lactate monitoring required. See LiMiT−AKI trial (27) and cohort study (28) for safety data. Proceed with caution.
SENP1-Sirt3 (26)	AMPK agonists	Remove SUMOylation and activate Sirt3	Mouse AKI model	SOD2 activity ↑, mitochondrial protection	Preclinical. Advantages:new mechanism. Challenges: requires specific agonists	Status: preclinical only, low evidence. Barriers: no specific agonist, unknown off−target effects. Applicability: hyperinflammatory phase, possibly prophylactic
Sirt3 (29)	NAD+ precursor (NMN)	Enhance Sirt3 activity	Cisplatin AKI model	DHODH stable ↑, ferroptosis ↓	Preclinical/Early Clinical. Advantages: NAD+ precursor. Challenges: broad systemic effects	Status: Phase I/II in aging/metabolic disease, no SA−AKI trial, low evidence. Barriers: systemic NAD^+^ may fuel tumors; narrow therapeutic window in sepsis. Applicability: older SA−AKI patients with low NAD^+^; not in active malignancy
NOX4 (30)	GLX7013114	Specific inhibition of NOX4; Activate Nrf2	IR−AKI mice	ROS ↓, mitochondrial function ↑, apoptosis ↓	Clinical exploration. Advantages: specific. Challenges: treatment window, possible stress reduction	Status: Phase I/II in diabetic kidney disease, no SA−AKI trial, low evidence. Barriers: treatment window <12 h, renal specificity not proven. Applicability: early SA−AKI with robust oxidative stress (e.g., high urinary H_2_O_2_)
NOX4 (31)	rIPC	Inhibition of NOX4-ROS pathway	Animal models/clinical exploration	Oxidative stress ↓, mitochondrial protection	Preclinical. Advantages: non-pharmacological, good safety. Challenges: mechanism needs verification	Status: preclinical only, small cardiac surgery AKI studies (low evidence). Barriers: difficult in unstable septic patients; unknown optimal timing. Applicability: prophylactic in high−risk surgical patients; not for established septic shock
Drp1-Fis1 (32)	P110 peptide	Specific blockade of Drp1−Fis1 interaction	CLP mice; LPS cells	Mitochondrial fragmentation ↓, renal function ↑	Preclinical. Advantages: high specificity, avoids comprehensive Drp1 inhibition. Challenges: peptide stability/delivery	Status: preclinical only, low evidence. Barriers: peptide stability, renal tubule delivery, immunogenicity. Applicability: early intervention (<6 h) in hyperdynamic sepsis; not for late phase
LRRK2 (33)	LRRK2 kinase inhibitor	Inhibition of MFN2 phosphorylation degradation	AKI mouse model	MFN2 stable ↑, fusion recovery	Preclinical. Advantages: novel. Challenges: kidney specificity needed to avoid neurological side effects	Status: preclinical only (Parkinson’s trials exist but not for SA−AKI), low evidence. Barriers: off-target lung/immune effects; narrow window. Applicability: SA−AKI with pre-existing mitochondrial fragmentation (biomarker−guided)
Ferroptosis (29)	MitoQ	Mitochondrial targeted antioxidant; Restore DHODH function	Cisplatin AKI model	CoQH_2_ ↑, lipid peroxidation ↓	Preclinical/clinical (other indications). Advantages: repositioning potential. Challenges: renal tubule delivery, short half-life	Status: Phase II in CKD/Parkinson’s, no SA-AKI trial, low evidence. Barriers: insufficient renal delivery; short half-life. Applicability: SA−AKI with iron overload/hemolysis (e.g., post-transfusion); early phase
HUWE1 (34)	HUWE1 inhibitor	Reduce MUTYH degradation; Maintain mtDNA repair	AKI mouse model	mtDNA damage ↓, inflammation ↓	Preclinical. Advantages: new target. Challenges: requires specific inhibitors	Status: preclinical only, low evidence. Barriers: no available inhibitor; potential off−target on cell cycle. Applicability: mtDNA-driven inflammation (e.g., high plasma mtDNA); precision medicine
cGAS (35)	cGAS inhibitor (RU.521)	Blocking cGAS recognition of mtDNA	AKI related research	Inflammation ↓, fibrosis ↓	Preclinical. Advantages: blocks upstream. Challenges: renal targeting needed to avoid infection risk	Status: preclinical only, few *in vivo* studies, low evidence. Barriers: infection risk (impaired pathogen sensing); renal delivery. Applicability: hyperinflammatory SA-AKI with proven mtDNA−cGAS activation; short-term use
STING (35)	STING inhibitor (H-151)	Block downstream signals of STING	AKI related research	Inflammatory factors ↓, renal injury ↓	Preclinical. Advantages: downstream blockade. Challenges: renal targeting, infection risk	Status: preclinical only, low evidence. Barriers: similar infection risk; long−term safety unknown. Applicability: same as cGAS; potentially wider window because downstream of multiple pathways

#### 3.1.2 Sterol regulatory element-binding protein 1c and YME1 like 1 ATPase

SREBP1c represents a transcriptional link between inflammation and lipid dysregulation. Although its direct role in SA-AKI awaits confirmation, studies in related AKI models demonstrate that SREBP1c represses YME1L1, an inner mitochondrial membrane protease essential for maintaining cristae structure and FAO. Given that inflammation potently induces SREBP1c and its downstream consequences—mitochondrial dysfunction and lipotoxicity—closely mirror SA-AKI pathology, the SREBP1c/YME1L1 axis emerges as a compelling candidate mechanism warranting further investigation (36).We propose this axis as a hypothesis-generating observation that requires direct testing in SA-AKI models.

#### 3.1.3 CD44

CD44 provides a direct route from extracellular inflammatory signals to intracellular metabolic control. In Lipopolysaccharide (LPS)-induced SA-AKI, CD44 is upregulated in tubular epithelial cells and promotes nuclear translocation of NF-κB p65. NF-κB p65 then directly binds and represses the Peroxisome proliferator-activated receptor gamma coactivator 1-alpha (PGC-1α) promoter. This suppresses mitochondrial biogenesis and FAO, precipitating energy crisis and oxidative stress. Tubule-specific CD44 deletion disrupts this vicious cycle and attenuates renal injury (37).

#### 3.1.4 Chemokine ligand 1

CX3CL1 contributes to mitochondrial injury primarily through immune-mediated mechanisms. By activating the Wnt/β-catenin pathway, CX3CL1 exacerbates macrophage infiltration and renal inflammation. CX3CL1 knockdown not only reduces inflammatory damage but also improves mitochondrial morphology and upregulates biogenesis proteins (PGC-1α, MFN2, uncoupling protein 2), suggesting that targeting this chemokine may create a permissive environment for mitochondrial recovery. However, GongQ et al.’s research only focused on podocytes. Whether and how CX3CL1 directly regulates the mitochondrial quality control procedures of renal tubular epithelial cells remains to be directly verified by future research (38).

#### 3.1.5 NADPH oxidase

NOX4 is a primary source of pathological ROS in SA-AKI. Upregulated in tubular cells during sepsis, NOX4-generated ROS directly attack mitochondria, causing loss of membrane potential, morphological abnormalities, and ATP depletion. Simultaneously, ROS activate NF-κB, amplifying cytokine production and establishing a feed-forward loop of injury (39). Genetic deletion or pharmacological inhibition of NOX4 (e.g., with GLX7013114) preserves mitochondrial function and reduces tubular damage (30). Notably, remote ischemic preconditioning (rIPC) confers renal protection by suppressing the NOX4-ROS pathway, positioning NOX4 as both a therapeutic target and a potential efficacy biomarker (31).

Although classical rIPC is applied before injury and thus has limited direct applicability to patients with established sepsis, emerging evidence indicates that rIPC is not restricted to the pre-injury window. A delayed protective phase (24–48 hours after application) exists (40), and delayed rIPC has been shown to protect against septic AKI in preclinical studies (41). Moreover, remote ischemic post-conditioning has been reported to be beneficial in animal models of septic shock (42), and an ongoing clinical trial (RIPC-ICU, NCT05830669) is evaluating rIPC in septic patients enrolled within 12 hours of diagnosis (43). Thus, rIPC remains a promising preventive strategy for early sepsis patients at risk of developing AKI, rather than a treatment for established SA-AKI.

### 3.2 Mitochondrial synthesis and metabolism

The kidney, particularly the proximal tubule, has high energy demands to support active transport, relying heavily on mitochondrial oxidative phosphorylation and FAO. In SA-AKI, this energetic machinery is severely compromised through multiple interconnected mechanisms, leading to bioenergetic failure that underpins tubular injury.

#### 3.2.1 Impaired mitochondrial biogenesis

##### 3.2.1.1 PGC-1α

PGC-1α, the master transcriptional coactivator of mitochondrial biogenesis, is generally downregulated in the established phase of SA-AKI (10, 24). While the temporal dynamics of PGC-1α expression may be complex and some studies have suggested potential early changes, the preponderance of evidence indicates that persistent PGC-1α decline is a central event driving mitochondrial dysfunction in SA-AKI. Reduced PGC-1α leads to decreased expression of nuclear respiratory factor 1 and 2 (NRF-1/2) and mitochondrial transcription factor A (TFAM), resulting in insufficient mitochondrial production and reduced mtDNA copy number. Concurrently, PGC-1α downregulation diminishes antioxidant enzyme expression (e.g., superoxide dismutase 2, glutathione peroxidase), weakening cellular defense against oxidative stress. Critically, it also suppresses key FAO enzymes such as Carnitine palmitoyltransferase 1A (CPT1A), forcing cells to rely on inefficient glycolysis and exacerbating the energy deficit. This creates a vicious cycle wherein dysfunctional mitochondria generate more ROS, further suppressing PGC-1α and deepening the bioenergetic crisis (10).

#### 3.2.2 Defective substrate utilization

CPT1A is the rate-limiting enzyme for mitochondrial FAO, a pathway essential for meeting the high energy demands of proximal tubular cells. In SA-AKI, CPT1A expression and function are markedly inhibited, largely due to the collapse of PGC-1α-driven transcriptional programs. This impairment prevents long-chain fatty acids from entering the mitochondria for oxidation, producing a dual insult: on one hand, cells experience severe “energy bankruptcy” with sharply reduced ATP production; on the other, unoxidized fatty acids accumulate intracellularly, generating lipotoxic species that directly damage mitochondrial membranes and trigger inflammatory and cell death pathways. Restoring CPT1A function therefore represents a direct strategy to correct pathogenic metabolic reprogramming and preserve mitochondrial integrity (10).

#### 3.2.3 Metabolic enzyme dysregulation

Beyond defects in biogenesis and substrate utilization, the function of key mitochondrial metabolic enzymes themselves is compromised in SA-AKI through post-translational modifications.

##### 3.2.3.1 Aldehyde dehydrogenase 2

ALDH2 exemplifies how metabolite-driven modifications can precisely regulate mitochondrial function. Under basal conditions, ALDH2 promotes mitochondrial biogenesis by deacetylating and activating PGC-1α, serving a protective role during early stress (44). However, in SA-AKI, accumulated lactate induces lactylation of ALDH2 at the K52 site. This modification causes aberrant nuclear translocation of ALDH2, where it interferes with the transcription of Prohibitin 2 (PHB2) — a critical mitophagy receptor — thereby disrupting clearance of damaged mitochondria and exacerbating injury (45). The specificity of anti-lactyl-lysine antibodies was validated in the original study using orthogonal approaches, including competitive peptide blocking, site-directed mutagenesis (K52R), and parallel mass spectrometry. Moreover, the protective effect of the K52R mutation was demonstrated in two distinct AKI models (cisplatin-induced and ischemia-reperfusion injury) within the same study, providing cross-model validation (45). To date, this mechanism has been primarily characterized by a single research group; further independent validation would be valuable. Thus, the functional shift of ALDH2 from protector to damage amplifier represents a key molecular switch in SA-AKI.

##### 3.2.3.2 Dihydroorotate dehydrogenase

DHODH is a multifunctional enzyme located in the inner mitochondrial membrane, involved in both pyrimidine synthesis and maintenance of redox balance. Under the intense oxidative stress characteristic of AKI, Sirt3 is inactivated by SUMOylation and cannot deacetylate and stabilize DHODH. Acetylated DHODH is subsequently degraded, triggering a cascade of consequences: reduced production of the lipophilic antioxidant coenzyme QH_2_ (CoQH_2_) collapses the cellular antioxidant barrier, while global mitochondrial metabolism becomes dysregulated (29). Together, these events promote lipid peroxidation and ultimately trigger ferroptosis in tubular epithelial cells. The Sirt3-DHODH-CoQH_2_ axis therefore represents a critical juncture where redox imbalance escalates into irreversible cell death.

### 3.3 Mitochondrial dynamics and quality control imbalance

Mitochondrial homeostasis depends on a delicate balance between fission and fusion (dynamics) and the selective clearance of damaged organelles via mitophagy (quality control). In SA-AKI, both arms of this maintenance system are disrupted, leading to progressive accumulation of dysfunctional mitochondria that perpetuate cellular injury.

#### 3.3.1 Dynamics imbalance: fragmentation prevails over fusion

Drp1 (dynamin-related protein 1) is the core executor of mitochondrial fission. In SA-AKI, Drp1 is hyperactivated, driving excessive mitochondrial fragmentation. A key mechanism involves lactate accumulated during metabolic reprogramming: lactate induces lactylation of Mitochondrial fission protein 1 (Fis1) at the K20 site, a major Drp1 receptor on the outer mitochondrial membrane. This modification enhances the Fis1-Drp1 interaction, leading to aberrant Drp1 recruitment and over-activation (32). The resulting fragmentation disrupts cristae structure, impairs oxidative phosphorylation, and generates fragmented mitochondria that are more prone to mtDNA leakage and ROS production. Targeting the Drp1-Fis1 interaction with specific inhibitory peptides (e.g., P110) effectively attenuates mitochondrial fragmentation and renal injury in experimental models. Although Drp1 is essential for healthy mitochondrial division and quality control, the P110 peptide is designed as a selective inhibitor of the pathological Drp1-Fis1 interaction without blocking Drp1 binding to other adaptors such as Mff, which is required for physiological fission (32). Consistent with this selectivity, P110 treatment has minimal effects on basal mitochondrial morphology and cell viability, and high-dose administration in AKI models showed no apparent toxicity (32). Nevertheless, as with any fission inhibitor, the timing and duration of intervention must be carefully considered. In the context of SA-AKI, the therapeutic window likely lies in acute, short-term inhibition during the early phase of excessive fission, rather than chronic suppression that could compromise mitophagy. Further studies are needed to define the optimal dosing regimen and to evaluate long-term safety.

Parallel pathways also contribute to fission activation. RCAN1 (regulator of calcineurin 1), an endogenous modulator of calcium signaling, is upregulated in AKI and activates the c-Jun N-terminal kinase (JNK) kinase pathway. JNK phosphorylates Mff (mitochondrial fission factor), enhancing its ability to recruit Drp1 and promoting excessive division (46). RCAN1 deletion alleviates this pathological fission and reduces apoptosis, highlighting the RCAN1-JNK-Mff axis as an additional target for intervention.

While fission is pathologically enhanced, fusion is simultaneously impaired. Leucine-rich repeat kinase 2 (LRRK2), activated in AKI, phosphorylates and activates the Mitogen-activated protein kinase kinase 4 (MKK4)/JNK pathway, which specifically targets MFN2 (mitofusin 2)—a key protein mediating outer mitochondrial membrane fusion. Phosphorylation of MFN2 at Ser27 triggers its ubiquitination and degradation, crippling the cell’s ability to repair damaged mitochondria through fusion (33). This LRRK2-driven MFN2 loss tilts the dynamic balance irreversibly toward fragmentation, independent of the canonical PTEN-induced putative kinase 1(PINK1)/Parkin RBR E3 ubiquitin protein ligase (Parkin) pathway.

#### 3.3.2 Quality control collapse: failed clearance of damaged mitochondria

Mitophagy, the selective autophagic clearance of dysfunctional mitochondria, serves as a critical quality control mechanism. In SA-AKI, multiple nodes within this pathway are compromised.

Sirt3, the primary NAD^+^-dependent deacetylase in mitochondria, plays a central role in maintaining mitochondrial homeostasis. In SA-AKI, Sirt3 is inactivated through multiple mechanisms: its expression is suppressed by macrophage-derived exosomal miR-195a-5p (47), and its activity is impaired by stress-induced SUMOylation (26, 29). The specific E3 SUMO ligase responsible for this modification in SA-AKI remains to be identified. However, the deSUMOylase SENP1 is a well-characterized regulator of this process; AMPK activation upregulates SENP1, which removes SUMO modifications from Sirt3 and restores its activity (26). Therefore, rather than targeting the unidentified E3 ligase, activating the AMPK-SENP1-Sirt3 axis (e.g., via metformin) represents a more immediate therapeutic strategy to restore Sirt3 function, as detailed in **Table 2**. Sirt3 inactivation triggers a cascade of failures: (1) reduced deacetylation of SOD2 impairs ROS clearance; (2) failure to activate FAO enzymes exacerbates energy crisis; (3) loss of DHODH stabilization (see Section 2.2.3) couples metabolic defects to ferroptosis (29). Restoring Sirt3 activity via AMPK agonists (e.g., metformin) or NAD^+^ precursors (e.g., NMN) has shown protective potential.

SENP1 acts upstream of Sirt3 by removing inhibitory SUMO modifications. AMPK activation upregulates SENP1, which deSUMOylates and reactivates Sirt3, restoring its deacetylase function (26). The AMPK-SENP1-Sirt3 axis thus represents an endogenous protective pathway that can be therapeutically augmented.

Mitophagy receptor proteins are also directly targeted. FUN14 domain-containing protein 1 (FUNDC1), an outer mitochondrial membrane receptor that initiates mitophagy in response to hypoxia, is regulated by a phosphorylation code: under basal conditions, Src and casein kinase 2(CK2) kinases phosphorylate FUNDC1 at Tyr18 and Ser13, inhibiting microtubule-associated protein 1A/1B-light chain 3 (LC3) binding; under stress, unc-51 like autophagy activating kinase 1(ULK1) phosphorylates Ser17 while phosphoglycerate mutase family member 5(PGAM5) dephosphorylates Ser13, promoting mitophagy (48). In SA-AKI, inflammatory and oxidative stress may disrupt this delicate balance, impairing FUNDC1 activation and allowing damaged mitochondria to accumulate.

**Figure 2:**
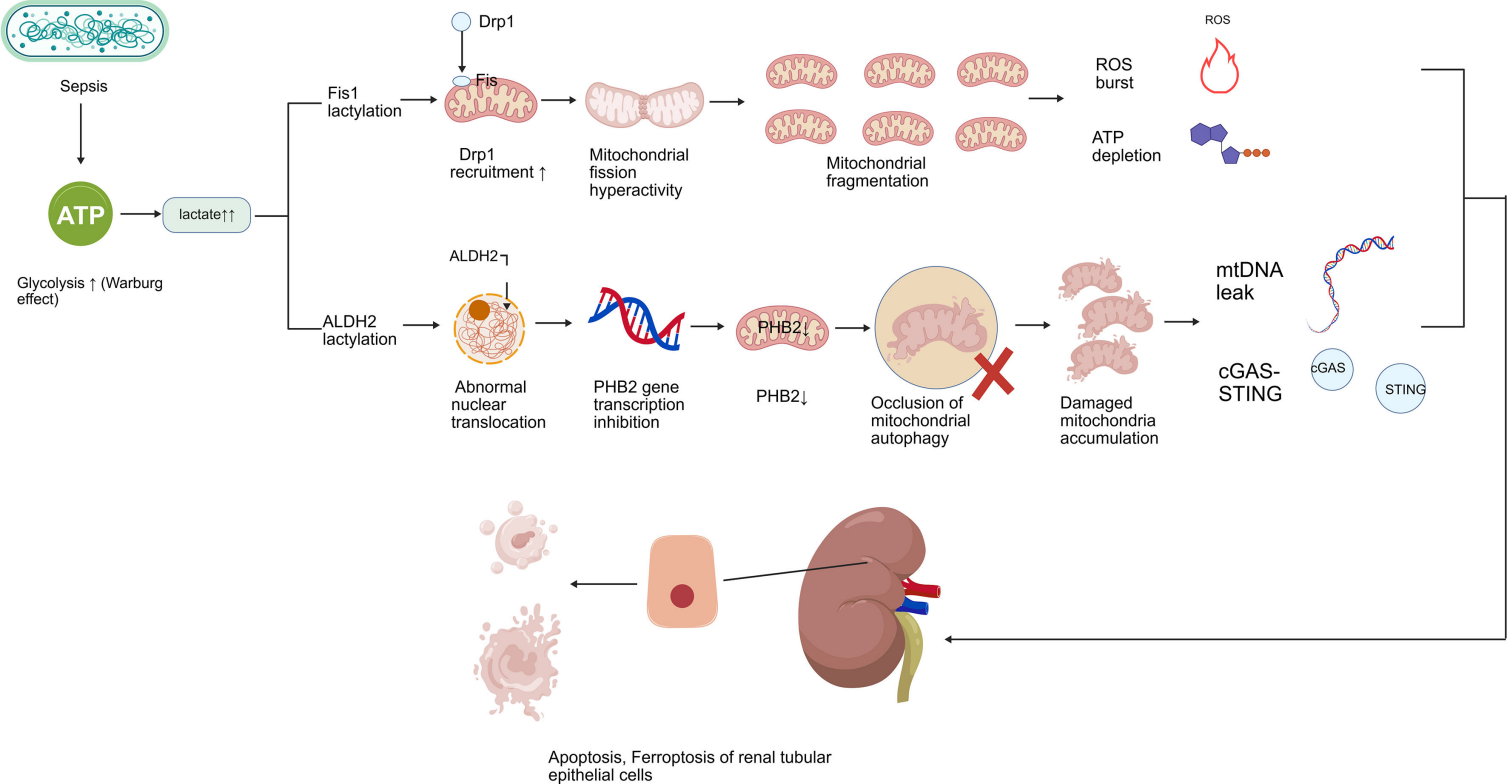
Lactylation-driven mitochondrial dysfunction in SA-AKI. Lactate accumulated during metabolic reprogramming drives lactylation of key mitochondrial proteins. Fis1 K20 lactylation enhances Drp1 binding, promoting excessive mitochondrial fragmentation. ALDH2 K52 lactylation causes its aberrant nuclear translocation, where it suppresses PHB2 transcription, impairing mitophagy. These parallel pathways converge on tubular epithelial cell injury. The **Figure 2** was created with BioGDP (9).

PHB2, an inner mitochondrial membrane protein, serves as a back-up mitophagy receptor when outer membrane integrity is compromised. Recent studies reveal that lactylated ALDH2 translocates to the nucleus and interferes with PHB2 transcription, leading to reduced PHB2 expression (46). This loss dismantles the last line of mitophagic defense, preventing clearance of severely damaged mitochondria with ruptured outer membranes. The resulting accumulation of these organelles amplifies ROS production and mtDNA release, fueling inflammation and cell death. **Figure 2** shows Lactylation-Driven Mitochondrial Dysfunction in SA-AKI.

### 3.4 Mitochondrial-organelle crosstalk and ion homeostasis

Mitochondria do not function in isolation but are intimately connected with other cellular organelles, particularly the endoplasmic reticulum (ER), through specialized contact sites. These interactions regulate critical processes including calcium transfer, lipid metabolism, and mitochondrial dynamics. In SA-AKI, disruption of this inter-organelle communication—especially calcium homeostasis—has emerged as a key driver of mitochondrial damage and cell fate determination.

#### 3.4.1 Pathological calcium signaling: ER-mitochondrial calcium overload

##### 2.4.1.1 Pannexin 1

Panx1, a large-pore channel protein located in the ER membrane, has been identified as a critical initiator of calcium dysregulation in AKI. Under stress conditions, Panx1 is activated as a calcium leakage channel, causing aberrant depletion of the ER calcium store. The released calcium is rapidly taken up by adjacent mitochondria via ER-mitochondria contact sites (mitochondria-associated membranes, MAMs), resulting in mitochondrial calcium overload (49). This overload triggers opening of the mitochondrial permeability transition pore (mPTP), impairs electron transport chain function, and induces a burst of ROS production, culminating in severe mitochondrial dysfunction and energy crisis. The Panx1-mitochondrial calcium overload axis thus represents a central mechanism linking acute stress to persistent organellar dysfunction and subsequent tubular injury.

##### 2.4.1.2 Transient receptor potential ankyrin 1

TRPA1, a non-selective cation channel activated by inflammatory and oxidative metabolites, provides another route for calcium dysregulation. In cisplatin-induced AKI, TRPA1 activation promotes ER stress and disrupts MAM integrity, leading to aberrant calcium transfer to mitochondria (50). Calcium-overloaded mitochondria undergo membrane potential collapse, ROS burst, and ATP synthesis failure, ultimately triggering tubular epithelial cell apoptosis. Although this mechanism awaits direct demonstration in SA-AKI models, the abundance of TRPA1-activating metabolites (e.g., reactive oxygen species, inflammatory mediators) in the septic milieu makes it a compelling candidate pathway warranting investigation.

#### 3.4.2 Protective calcium signaling: activation of adaptive responses

In contrast to the deleterious effects of calcium overload, controlled calcium signals can activate adaptive cellular responses. TRPM2 (transient receptor potential melastatin 2), a calcium-permeable channel highly sensitive to oxidative stress, exemplifies this duality. In cisplatin-induced AKI, TRPM2 is activated by ROS and mediates mild, sustained calcium influx. Unlike the toxic calcium overload mediated by Panx1 or TRPA1, TRPM2-driven calcium signals function as second messengers, activating AKT (protein kinase B) and subsequently enhancing autophagic flux via the mammalian target of rapamycin (mTOR) pathway (51).

Enhanced autophagy facilitates timely clearance of protein aggregates and dysfunctional organelles—including damaged mitochondria—thereby maintaining cellular homeostasis and limiting injury progression.

The TRPM2-Ca²^+^-AKT-autophagy axis thus constitutes an important endogenous defense pathway. This mechanism stands in stark contrast to pathological calcium overload channels, highlighting the central role of calcium “dosage” and “spatiotemporal specificity” in determining cell fate under stress.

#### 3.4.3 Therapeutic implications

The dual nature of calcium signaling in SA-AKI presents both challenges and opportunities for therapeutic intervention. Strategies aimed at blocking pathological calcium channels (e.g., Panx1, TRPA1) may prevent mitochondrial calcium overload and subsequent injury, while preserving or enhancing protective calcium signals (e.g., through TRPM2) could boost endogenous repair mechanisms. However, the temporal and spatial complexity of calcium signaling demands precise targeting to avoid unintended disruption of physiological calcium homeostasis.

We note that the role of TRPM2 is context−dependent and differs across organs. While TRPM2 activation promotes autophagy and limits tubular injury in cisplatin−induced AKI via the Ca^2+^-AKT-mTOR pathway (51), TRPM2 overactivation in the brain contributes to neuronal death and blood−brain barrier disruption in ischemic stroke (52, 53). This organ-specific difference may be attributed to distinct downstream signaling pathways and cell−type expression patterns. In the kidney, the protective effect of TRPM2 is not achieved by hyperactivation, but rather by preserving its physiological signaling tone that supports adaptive autophagy. Moreover, as TRPM2 in renal I/R injury has been shown to localize mainly to proximal tubular epithelial cells rather than hematopoietic cells (14), kidney−specific targeting strategies could theoretically avoid systemic adverse effects. Systemic TRPM2 activation is not proposed in this review; rather, we emphasize the importance of maintaining endogenous protective calcium signaling within a physiological range, as discussed above regarding calcium “dosage and spatiotemporal specificity”.

### 3.5 Mitochondrial DNA damage and innate immune activation

Mitochondrial DNA is particularly vulnerable to oxidative damage due to its proximity to the electron transport chain, lack of protective histones, and limited repair capacity. When damaged, mtDNA not only impairs mitochondrial function but also acts as a potent damage-associated molecular pattern (DAMP) when released into the cytoplasm or extracellular space, linking mitochondrial damage directly to innate immune activation as introduced in Section 1.1. In SA-AKI, the dual consequence of mtDNA damage—bioenergetic compromise and immune activation—creates a vicious cycle that amplifies renal injury.

#### 3.5.1 mtDNA damage and repair failure

##### 3.5.1.1 MutY DNA glycosylase

MUTYH is a key enzyme in the base excision repair pathway, specifically recognizing and excising 8-oxoguanine—a hallmark product of oxidative DNA damage—thereby maintaining mtDNA integrity. In AKI, MUTYH function is compromised through upstream mechanisms. Studies demonstrate that the E3 ubiquitin ligase -HECT, UBA and WWE domain containing E3 ubiquitin protein ligase 1(HUWE1) is upregulated and directly binds to MUTYH, catalyzing its ubiquitination and subsequent proteasomal degradation (34).

Loss of MUTYH prevents timely repair of oxidative mtDNA damage, leading to accumulation of mutations and reduction in mtDNA copy number. Critically, damaged mtDNA is more prone to leakage from mitochondria into the cytoplasm, where it is recognized by pattern recognition receptors such as cyclic GMP-AMP synthase(cGAS), activating innate immune pathways. The HUWE1-MUTYH axis thus represents a key link connecting oxidative stress to mitochondrial inflammation, and stabilizing MUTYH or inhibiting HUWE1 hyperactivation may represent novel therapeutic strategies.

#### 3.5.2 Safeguarding mitochondrial membrane integrity

Preventing mtDNA leakage requires intact mitochondrial membranes. DUSP1 (dual specificity phosphatase 1), a negative regulator of MAPK signaling, has emerged as a critical protector of mitochondrial genome stability and innate immune homeostasis.

Under stress conditions such as ischemia, DUSP1 expression is upregulated. It exerts its protective effects by directly dephosphorylating and inhibiting JNK kinase activity, thereby blocking the translocation and activation of the pro-apoptotic protein BCL2-associated X (BAX) to mitochondria (35). BAX activation is a key step leading to increased outer mitochondrial membrane permeability and mtDNA release. By inhibiting the JNK/BAX axis, DUSP1 effectively reduces mtDNA leakage into the cytoplasm.

Leaked mtDNA is a potent activator of the cGAS-(Stimulator of interferon genes)STING pathway. Upon binding mtDNA, cGAS generates 2’3’-cyclic GMP-AMP (2’3’-cGAMP), which activates STING and triggers a robust inflammatory response including type I interferon production and NF-κB activation. DUSP1 deficiency exacerbates mtDNA leakage, cGAS-STING pathway activation, and subsequent renal fibrosis (35). The DUSP1-JNK-BAX-mtDNA axis therefore constitutes an important endogenous protective pathway that maintains mitochondrial membrane integrity at the source and prevents the propagation of damage signals that trigger inflammatory storms.

#### 3.5.3 Therapeutic implications

**Figure 3:**
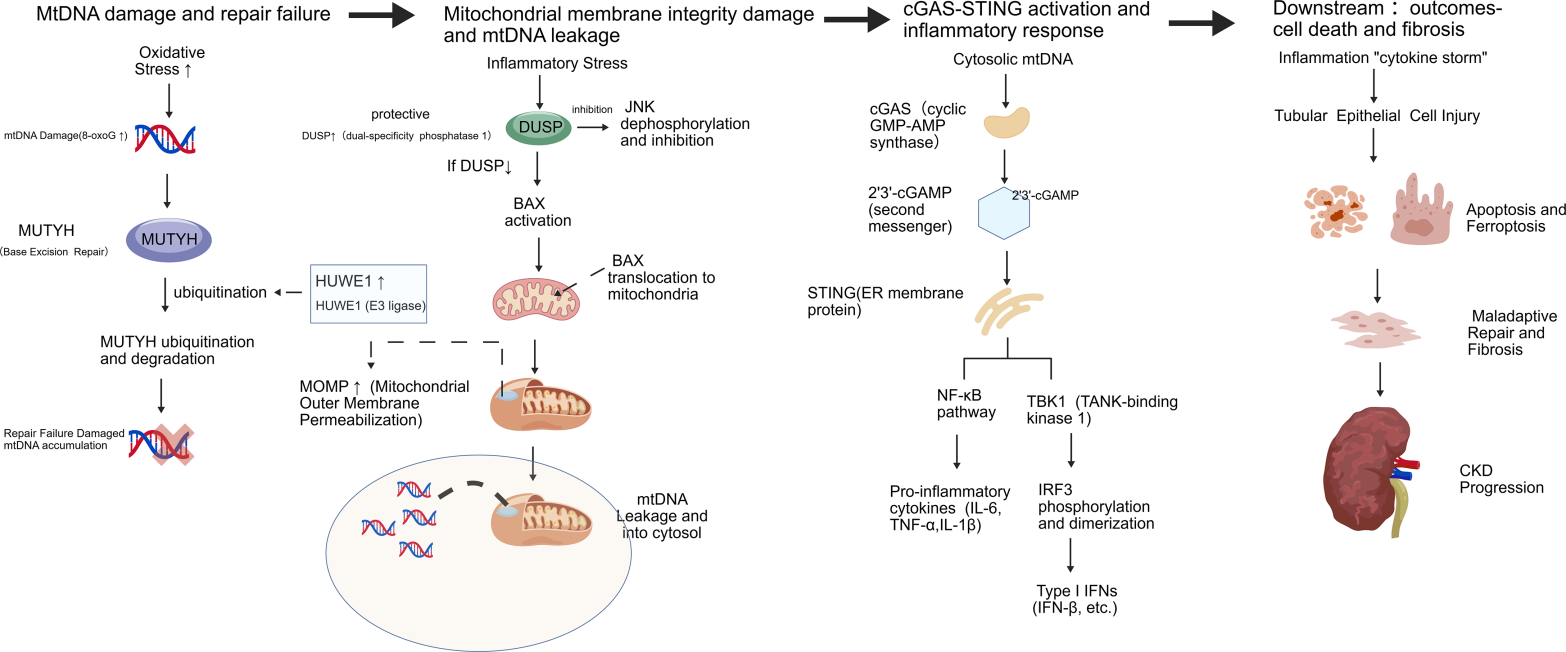
mtDNA damage and cGAS-STING-mediated innate immune activation in SA-AKI. A three-stage cascade: (1) Oxidative stress induces mtDNA damage (8-oxoG), but repair enzyme MUTYH is degraded by HUWE1, leading to damage accumulation. (2) DUSP1 inhibits JNK/BAX axis to preserve mitochondrial membrane integrity; DUSP1 deficiency allows BAX-mediated MOMP and mtDNA leakage. (3) Cytosolic mtDNA activates cGAS-STING pathway, triggering TBK1/IRF3 and NF-κB signaling, resulting in inflammation, cell death, and fibrosis. The **Figure 3** was created with BioGDP (9).

The recognition of mtDNA as a key immunostimulatory molecule in SA-AKI opens new therapeutic avenues (54). Strategies aimed at preserving mtDNA integrity (e.g., enhancing MUTYH stability, inhibiting HUWE1), preventing mtDNA release (e.g., reinforcing DUSP1 function, stabilizing mitochondrial membranes), or blocking the sensing of leaked mtDNA (e.g., cGAS or STING inhibitors) could disrupt the self-perpetuating cycle of mitochondrial damage and inflammation (35, 55). However, the physiological role of mtDNA-mediated innate immunity in pathogen defense necessitates careful targeting to avoid compromising host defense against infection (54). It is also important to recognize that mtDNA-mediated inflammation is not limited to tubular epithelial cells. Infiltrating macrophages and dendritic cells also experience mitochondrial dysfunction during sepsis and release mtDNA, which contributes to the systemic inflammatory response and amplifies renal injury. Conversely, mitochondrial-targeted therapies that reduce mtDNA leakage in immune cells may have dual benefits: dampening systemic inflammation while directly protecting the kidney. Future studies should dissect the cell-type-specific contributions of mtDNA release to SA-AKI pathogenesis, as this may inform the development of targeted immunomodulatory strategies. **Figure 3** shows mtDNA Damage and cGAS-STING-Mediated Innate immune activation in SA-AKI. **Table 3** shows the key molecular mediators of mitochondrial dysfunction in SA-AKI.

**Table 3:** Key molecular mediators of mitochondrial dysfunction in SA-AKI.

Molecular	Classification	Subcellular localization	Changes in SA-AKI	Core functions/mechanisms	Intervention potential	References
AMPK	Upstream signal	Cytoplasm/mitochondria	Activity ↓	Metabolic sensor; activates SENP1-Sirt3 axis; suppresses NF-κB	Metformin activation	(26)
SREBP1c	Upstream signal	Nucleus	Expression ↑	Transcriptional inhibition of YME1L1; causes damage to mitochondrial structure	Yet to be verified	(36)
CD44	Upstream signal	Cell membrane	Expression ↑	Promote nuclear translocation of NF-κ Bp65; inhibit PGC-1α transcription	Gene knockout protection	(37)
NOX4	Upstream signal	Mitochondria/Endoplasmic reticulum	Activity ↑	Generation of ROS; direct damage to mitochondria; activation of NF-κB	GLX7013114 RIPC	(30, 31, 39)
PGC-1α	Biosynthesis	Nucleus	Expression ↓	Main regulator of mitochondrial biosynthesis; regulates antioxidant enzymes and FAO	Activator	(10)
CPT1A	Metabolism	Outer Mitochondrial membrane	Function ↓	FAO rate-limiting enzyme; ensuring mitochondrial energy supply	Functional recovery	(10)
ALDH2	Metabolism/Quality Control	Mitochondria/Nucleus	Lactylation ↑	Protective (activates PGC-1α) → injurious (nuclear translocation inhibits PHB2)	Inhibitory lactic acid	(44)
DHODH	Metabolism	Inner mitochondrial membrane	Acetylation → Degradation	Maintain CoQH_2_ synthesis; resist oxidation; inhibit iron death	MitoQ, NMN	(29)
Drp1	Dynamics	Cytoplasm/Mitochondria	Activate ↑	Core executor of mitochondrial division; excessive activation leads to fragmentation	P110 peptide inhibitors	(32)
Fis1	Dynamics	Outer Mitochondrial membrane	K20 lactylation ↑	Drp1 receptor; lactylation enhances Drp1 binding	Targeting interaction	(32)
LRRK2	Dynamics	Cytoplasmic	Activate ↑	Phosphorylated MFN2 promotes its degradation; inhibits fusion	Kinase inhibitor	(33)
MFN2	Dynamics	Outer Mitochondrial membrane	p-Ser27 degradation	Outer Mitochondrial membrane fusion protein; degradation leads to fusion defects	Stabilize MFN2	(33)
Sirt3	Quality control	Mitochondrial matrix	Expression ↓/SUMOylation ↑	NAD^+^-dependent deacetylase; regulates SOD2, FAO, DHODH	NAD ^+^ precursor (NMN)	(26, 29, 47)
FUNDC1	Quality control	Outer Mitochondrial membrane	Regulation imbalance	Hypoxia-induced mitochondrial autophagy receptor; regulated by phosphorylation	Activate autophagy	(48)
PHB2	Quality control	Inner mitochondrial membrane	Expression ↓	Inner membrane autophagy receptor; transcription suppressed by ALDH2 lactylation-inhibition	Restore expression	(44)
MUTYH	Mitochondrial repair	Mitochondrial matrix	Protein degradation	8-oxoG repair enzyme; ubiquitinated and degraded by HUWE1	Stable MUTYH	(34)
DUSP1	Mitochondrial immunity	Cytoplasmic	Expression ↑ (protective)	Inhibit JNK/BAX; reduce mtDNA leakage; suppress cGAS-STING	Enhanced expression	(35)
cGAS	Mitochondrial immunity	Cytoplasmic	Activation	DNA sensor; recognition of cytoplasmic mtDNA; activation of STING	cGAS inhibitors	(35)
STING	Mitochondrial immunity	Endoplasmic reticulum membrane	Activation	Mediates downstream inflammatory signals; TBK1/IRF3 and NF-κB	STING inhibitor	(35)

The symbol “↓” indicates a decrease or downregulation, “↑” indicates an increase or upregulation, and “→” indicates a directional progression (e.g., leads to or results in).

In addition to the mtDNA-targeted approaches discussed above, **Table 2** summarizes a broader range of emerging therapeutic strategies targeting mitochondrial dysfunction in SA-AKI. To critically evaluate their clinical translatability, we have extended our analysis beyond mechanistic correspondence to include three key dimensions: current clinical study status, major translational barriers, and applicability in specific SA-AKI clinical contexts.

First, regarding clinical study status, most strategies remain at preclinical stages, with only a few having entered early-phase clinical trials in other disease settings (e.g., metformin in diabetes, MitoQ in chronic kidney disease, NMN in aging/metabolic disorders). However, no large-scale SA-AKI-specific trial has been reported for any of these approaches. The level of evidence is therefore uniformly low (for agents with Phase I/II data in other indications) or very low (for purely preclinical strategies). This highlights a major gap that requires dedicated SA-AKI trials.

Second, key translational barriers include safety concerns (e.g., lactic acidosis risk with metformin in septic shock; infection risk with cGAS/STING inhibitors), lack of renal tubule-specific targeting (e.g., MitoQ, P110 peptide), and narrow therapeutic windows (e.g., <12 h for NOX4 inhibitors; <6 h for Drp1-Fis1 blockade). Overcoming these barriers will likely require innovative delivery systems (e.g., nanoparticle-encapsulated agents) and careful patient selection based on biomarkers.

Third, applicability in specific SA-AKI contexts varies considerably. For example, STING or cGAS inhibitors may be most beneficial in the early hyperinflammatory phase with proven mtDNA release, but should be used short-term to avoid infection risk. In contrast, immunometabolic reprogramming agents (e.g., itaconate derivatives) or mitophagy enhancers (e.g., urolithin A) might better suit the late immunosuppressive phase characterized by mitochondrial paralysis in immune cells. For elderly patients or those with pre-existing chronic kidney disease, NAD^+^ precursors or mitophagy enhancers are preferable, whereas PGC-1α activators should be used with caution due to low mitochondrial reserve. In hemodynamically unstable septic shock, metformin is not recommended, and non-pharmacological interventions like rIPC are also of limited feasibility.

Collectively, this three-dimensional analysis — covering clinical study status, translational barriers, and context-specific applicability — elevates the discussion from mere mechanistic matching to a clinically oriented evaluation, thereby meeting the expected depth for a high-quality review on therapeutic strategies. The detailed information for each individual strategy is presented in **Table 2**.

## 4 Conclusions and future perspective

Mitochondrial dysfunction has emerged as a central pathophysiological hub in sepsis-associated acute kidney injury (SA-AKI), integrating signals from systemic inflammation, hemodynamic compromise, and metabolic reprogramming to dictate the fate of tubular epithelial cells (8). As reviewed herein, this dysfunction is not a monolithic entity but rather a multidimensional syndrome encompassing bioenergetic failure, dynamics imbalance, quality control collapse, aberrant organelle communication, and mtDNA-mediated innate immune activation (10).

Several unifying themes emerge from the growing body of evidence. First, post-translational modifications—particularly phosphorylation, acetylation, SUMOylation, and the recently recognized lactylation—have emerged as rapid and dynamic switches that fine-tune mitochondrial protein function in response to septic stress. The lactate-driven modifications of Fis1 (lactylation), ALDH2 (lactylation), and Drp1-interacting proteins exemplify how metabolic reprogramming directly impinges on mitochondrial dynamics and quality control (32). Second, mitochondria serve as signaling platforms that translate metabolic stress into immune activation, with mtDNA leakage activating the cGAS-STING pathway and creating a self-perpetuating cycle of inflammation and injury (54). Third, the dual nature of stress responses—exemplified by protective versus pathological calcium signaling (TRPM2 vs. Panx1/TRPA1) and the context-dependent roles of molecules like ALDH2—highlights the importance of dose, duration, and spatiotemporal context in determining cell fate (51).

Despite significant preclinical advances, translating these mechanistic insights into clinical practice faces considerable hurdles. Key challenges include:

Target specificity and timing: Many mitochondrial regulators have context-dependent dual roles. For example, Drp1-mediated fission is essential for healthy mitochondrial turnover but becomes pathogenic when hyperactivated; AMPK activation is protective in tubular cells but may have unintended effects in immune cells. Therapeutic interventions must therefore consider the precise timing, duration, and cell-type specificity of target modulation (7).

Biomarker development: Non-invasive biomarkers that reflect real-time mitochondrial health in the kidney are urgently needed to guide patient selection, monitor therapeutic response, and enable personalized medicine. Circulating mtDNA fragments, specific metabolic signatures, or urinary mitochondrial proteins may hold promise but require rigorous validation (56).

Heterogeneity of SA-AKI: The relative contribution of specific mitochondrial pathways may vary depending on sepsis etiology, stage of injury, and patient comorbidities. Stratifying patients based on dominant pathophysiological mechanisms will be essential for successful clinical translation (4).

Translation from AKI to CKD: Mitochondrial dysfunction persists beyond the acute phase and contributes to maladaptive repair and fibrosis. Understanding how acute mitochondrial injury transitions to chronic mitochondrial deficits may reveal windows for intervention that prevent progression to chronic kidney disease (10).

Future research directions should leverage emerging technologies to address these challenges. Single-cell and spatial transcriptomics can resolve the heterogeneity of mitochondrial responses across different nephron segments and immune cell populations (57). Advances in mitochondrial-targeted probes may enable real-time monitoring of mitochondrial function *in vivo* (58). Furthermore, the development of precision therapeutics—such as cell-permeable peptides that disrupt pathogenic protein-protein interactions (e.g., Drp1-Fis1), organelle-specific antioxidants, or gene-editing approaches to correct mtDNA damage—holds promise for mechanism-based intervention (55).The strategy-specific evaluation of clinical translatability presented in **Table 2** provides a roadmap for prioritizing these approaches in future SA-AKI trials.

The complexity of SUMOylation as a therapeutic target: SUMOylation is a highly context-dependent regulatory system. While Sirt3 SUMOylation inactivates this protective deacetylase, recent evidence demonstrates that SUMOylation of IκBα stabilizes the protein and suppresses NF-κB overactivation, thereby protecting against SA-AKI (59). This complexity highlights the need for precise, context-specific targeting strategies. Given that the specific E3 ligase for Sirt3 has not yet been identified, we suggest prioritizing the validated AMPK-SENP1-Sirt3 axis for therapeutic intervention. Future research should focus on delineating the precise roles of specific SUMOylation events and their regulatory enzymes in SA-AKI.

Based on the available evidence, we tentatively suggest a three-step roadmap to help prioritize future research efforts. First, for the near term (2–3 years), the mtDNA-cGAS-STING axis may be worth prioritizing. This suggestion is based on human biomarker data (elevated plasma/urinary mtDNA in SA-AKI patients), mechanistic studies linking mtDNA leakage to inflammasome activation, and the availability of STING/cGAS inhibitors that have already entered early-phase trials for other inflammatory diseases. Prospective cohort studies to validate urinary mtDNA as a biomarker, and proof-of-concept trials with STING inhibitors (e.g., H-151), could be considered. Second, for the mid-term (3–5 years), repurposed drugs with established safety profiles — such as metformin (LiMiT-AKI trial), NOX4 inhibitors, and MitoQ — could be evaluated in SA-AKI-specific randomized controlled trials. Third, for the longer term (5–10 years), emerging targets with promising preclinical data but significant translational barriers (e.g., P110 peptide due to delivery and stability issues; HUWE1 and ALDH2 inhibitors requiring further target validation) may continue to be explored in basic research and drug development. We offer this tentative prioritization in the hope of focusing resources on the most actionable targets while maintaining a pipeline of innovative approaches.

Ultimately, restoring mitochondrial homeostasis in SA-AKI will likely require combination strategies that simultaneously enhance biogenesis, rebalance dynamics, restore quality control, and limit inflammatory signaling. In summary, SA-AKI is not simply a disease of tubular energy failure but a syndrome of disrupted mitochondria-immune homeostasis. Therapeutic strategies that simultaneously restore mitochondrial function and dampen pathogenic innate immune signaling—rather than targeting either compartment alone—hold the greatest promise for breaking the self-perpetuating cycle of injury and inflammation. As our understanding of the mitochondrial pathophysiological network deepens, the integration of mitochondrial health assessment into the diagnostic and therapeutic framework for SA-AKI may transform the management of this devastating syndrome.

## Glossary

AKI: Acute kidney injury
ALDH2: Aldehyde dehydrogenase 2
AMPK: AMP-activated protein kinase
Ang II: Angiotensin II
AT1R: Angiotensin II type 1 receptor
ATP: Adenosine triphosphate
BAX: BCL2-associated X protein
CKD: Chronic kidney disease
CLP: Cecal ligation and puncture
CoQH_2_: Coenzyme QH_2_
CPT1A: Carnitine palmitoyltransferase 1A
CX3CL1: Chemokine (C-X3-C motif) ligand 1
DAMP: Damage-associated molecular pattern
DHODH: Dihydroorotate dehydrogenase
Drp1: Dynamin-related protein 1
DUSP1: Dual specificity phosphatase 1
ER: Endoplasmic reticulum
FAO: Fatty acid oxidation
Fis1: Mitochondrial fission protein 1
FUNDC1: FUN14 domain-containing protein 1
GSEA: Gene set enrichment analysis
HUWE1: HECT, UBA and WWE domain containing E3 ubiquitin protein ligase 1
IL-1β: Interleukin-1β
IL-6: Interleukin-6
IR: Ischemia-reperfusion
JNK: c-Jun N-terminal kinase
KDIGO: Kidney Disease: Improving Global Outcomes
LPS: Lipopolysaccharide
LRRK2: Leucine-rich repeat kinase 2
MAM: Mitochondria-associated membrane
Mff: Mitochondrial fission factor
MFN2: Mitofusin 2
MitoQ: Mitochondria-targeted ubiquinone
mPTP: Mitochondrial permeability transition pore
mtDNA: Mitochondrial DNA
MUTYH: MutY DNA glycosylase
NAD⁺: Nicotinamide adenine dinucleotide (oxidized form)
NF-κB: Nuclear factor kappa-light-chain-enhancer of activated B cells
NMN: Nicotinamide mononucleotide
NOX4: NADPH oxidase 4
NRF-1/2: Nuclear respiratory factor 1/2
PAMP: Pathogen-associated molecular pattern
Panx1: Pannexin 1
PGC-1α: Peroxisome proliferator-activated receptor gamma coactivator 1-alpha
PHB2: Prohibitin 2
PINK1: PTEN-induced putative kinase 1
PRR: Pattern recognition receptor
RAAS: Renin-angiotensin-aldosterone system
RCAN1: Regulator of calcineurin 1
rIPC: Remote ischemic preconditioning
ROS: Reactive oxygen species
SA-AKI: Sepsis-associated acute kidney injury
SENP1: Sentrin-specific protease 1
Sirt3: Sirtuin 3
SOD2: Superoxide dismutase 2
SREBP1c: Sterol regulatory element-binding protein 1c
STING: Stimulator of interferon genes
SUMO: Small ubiquitin-like modifier
TFAM: Mitochondrial transcription factor A
TNF-α: Tumor necrosis factor-α
TRPA1: Transient receptor potential ankyrin 1
TRPM2: Transient receptor potential melastatin 2
UCP2: Uncoupling protein 2
ULK1: Unc-51 like autophagy activating kinase 1
YME1L1: YME1 like 1 ATPase
